# The long-term outcomes of patients with negative prolonged ambulatory electroencephalography tests: A cross-sectional follow-up study

**DOI:** 10.12688/healthopenres.13351.1

**Published:** 2023-08-10

**Authors:** Sumika Ouchida, Armin Nikpour, Xin Zhang, Howard Faulkner, Maricar Senturias, Nicole Reid, Eleanor Stephens, Greg Fairbrother

**Affiliations:** 1 Department of Neurology, Royal Prince Alfred Hospital, Camperdown, New South Wales, 2050, Australia; 2 Faculty of Medicine and Health, The University of Sydney, Sydney, New South Wales, 2050, Australia; 3 Department of Neurology, Southmead Hospital, Bristol, BS10 5NB, UK; 4 Department of Neurology, Westmead Hospital, Westmead, New South Wales, 2145, Australia; 5 Sydney Reseach, Sydney Local Health District, Camperdown, New South Wales, 2050, Australia

**Keywords:** Ambulatory, Electroencephalography, Epilepsy, Diagnosis, Quality of life

## Abstract

**Background:**

Ambulatory electroencephalography (AEEG) recording is an essential aid for detecting interictal discharges and providing a clinical diagnosis. This study aimed to describe long-term outcomes among a cohort of patients who yielded negative results on AEEG at the time of assessment and identify factors associated with contemporary quality of life (QOL) and ultimate epilepsy diagnosis.

**Methods:**

This cross-sectional telephone follow-up study was conducted in June-November 2021 at the Neurology Department in a metropolitan hospital in Sydney, Australia.

**Results:**

In total, 47 of 105 eligible (45%) participants were enrolled. Overall, 21 (45%) participants had been diagnosed with epilepsy at a 12-year follow-up. Taking anti-seizure medication, having experienced a seizure event, and having marriage and education-related characteristics were associated with an epilepsy diagnosis. QOL was found to be associated with age, employment status and history of experience of a seizure event. QOL and an epilepsy diagnosis were not shown to be statistically related.

**Conclusions:**

Nearly half of the participants had received an epilepsy diagnosis at long-term follow-up, despite having tested negative on AEEG at the time of assessment. Prolonged AEEG testing is an important tool to aid the diagnostic process. However, clinical examination, including accurate history taking, is vital in establishing an epilepsy diagnosis.

## Plain language summary

Ambulatory electroencephalography (AEEG) recording is an essential aid for detecting interictal discharges and providing a clinical diagnosis. This study aimed to describe long-term outcomes among a cohort of patients who yielded negative results on AEEG at the time of assessment and identify factors associated with contemporary quality of life (QOL) and ultimate epilepsy diagnosis.

This cross-sectional telephone follow-up study was conducted in June-November 2021 at the Neurology Department in a metropolitan hospital in Sydney, Australia.

In total, 47 of 105 eligible (45%) participants were enrolled. Overall, 21 (45%) participants had been diagnosed with epilepsy at a 12-year follow-up. Taking anti-seizure medication, having experienced a seizure event, and having marriage and education-related characteristics were associated with an epilepsy diagnosis. QOL was found to be associated with age, employment status and history of experience of a seizure event. QOL and an epilepsy diagnosis were not shown to be statistically related.

Nearly half of the participants had received an epilepsy diagnosis at long-term follow-up, despite having tested negative on AEEG at the time of assessment. Prolonged AEEG testing is an important tool to aid the diagnostic process. However, clinical examination, including accurate history taking, is vital in establishing an epilepsy diagnosis.

## Introduction

 Ambulatory electroencephalography (AEEG) is a continuous EEG recording technique employed among patients in their natural settings outside the hospital-controlled environment. The recording devices have been improved in the last 50 years and are portable and cost-effective ^1– 3^. Recording inter-ictal epileptiform discharges or paroxysmal events using AEEG is an essential epilepsy diagnostic tool ^4– 7^. Prolonged AEEG is essential in assessing patients presenting with diagnostic or management difficulties. Faulkner *et al.* investigated the usefulness of prolonged AEEG tests ^7^. Their retrospective study analyzed the EEG data and clinical records associated with 324 consecutive prolonged AEEG tests lasting four to five days without medication withdrawal. More than two-thirds of patients in this study (219/324; 68%) had either paroxysmal events or EEG abnormalities during the four to five-day monitoring period. However, 32% (105/324) had not reported/recorded events or EEG abnormalities during the monitoring period. Among this sub-group, 30.5% (32/105) were not classified as having epilepsy based on clinical characteristics. Furthermore, 52.3% (55/105) took anti-seizure medication (ASM) during their tests. It should be noted that clinical and EEG data were reviewed. Participants' quality of life (QOL) was not measured.

 In our study, the aim was to assess the long-term outcome of patients without epileptiform features on their AEEG recordings on the basis of prospective follow-up rather than retrospective audit (as was done by Faulkner *et al.*) ^7^. Prolonged EEG recording is crucial for detecting interictal epileptiform discharges; however, the absence of discharges in prolonged EEG recording does not exclude a diagnosis of epilepsy ^8^. Approximately 10% of epilepsy patients never show epileptiform discharges on EEG tests ^9^. Some studies suggest that the QOL among people with epilepsy is low and that sociodemographic factors are associated with the QOL of people with epilepsy ^10, 11^. This study sought to describe long-term patient outcomes among this cohort and identify factors associated with QOL and epilepsy diagnosis.

## Methods

### Ethical approval

The study was approved by the Sydney Local Health District Human Research Ethics Committee (RPAH Zone) on 21 May 2021 (Protocol no: X21-0092 & 2021/ETH00545) and was performed in accordance with the ethical standards laid down in the 1964 Declaration of Helsinki and its later amendment.

### Study design

This cross-sectional telephone follow-up study was conducted from June to November 2021 at the Neurology Department of the Royal Prince Alfred Hospital in Sydney, Australia.

### Participants and consent

Consecutive patients (n=105) who underwent a four or five-day AEEG test between 2007 to 2010 without showing any epileptiform abnormalities on their prolonged EEG recordings were screened for study eligibility. This cohort of patients was identified in an earlier study ^4^. The earlier study examined adult participants aged 17–61 years with a seizure onset or event history from 1–46 years old. Patients were excluded if: i) they did not speak English; ii) they had cognitive impairment; iii) they were deceased; iv) they were deaf or mute. Verbal or written consent was obtained from the eligible participants. Verbal consent was obtained only when written consent was not received (this was due to discrepancy between email address and cell phone number), but telephone follow up indicated that participants wanted to be involved in the study. All consent processes were approved by the overseeing Ethics Committee.

### Study procedure

Before contacting eligible participants, a researcher checked the Death Register for each State to minimize the risk of contacting a deceased participant. A researcher or a research assistant then contacted eligible participants by telephone. The research team sent an information statement with a consent form by post and email if the participants expressed interest in the study. A quantitative telephone survey was conducted at a time suitable for consenting participants for a duration of 15–30 minutes. The survey gathered data on personal and clinical demographic characteristics and three areas of outcome-oriented interest: epilepsy diagnosis, physical QOL and mental QOL.

### Outcomes

 A key outcome of interest was the prevalence of ultimate epilepsy diagnosis among patients who had undergone a four to five-day AEEG test and had no evidence of epileptiform features or absence of reported events on their prolonged EEG recordings. The epilepsy diagnosis was made by asking participants: "Have you received an epilepsy diagnosis?" during the phone interview. If the participant said "yes" to the question, we asked: "What type of epilepsy do you have or have you been diagnosed with ?" A further outcome of interest was QOL, measured using the SF-12v2® questionnaire. The SF-12v2® is a short form of a health survey developed from the Medical Outcomes Study (MOS) 36-item Short-Form Health Survey (SF-36) ^12^. The SF-12v2® is one of the most widely used instruments for assessing self-reported health-related quality of life. The SF-12v2® covers the same eight health domains as the SF-36 with substantially fewer questions, making it a more practical research tool, especially among populations with limited attention spans or mental health problems ^13^. Aggregate QOL scores reflecting Physical QOL and Mental QOL are commonly derived. These summary scores have been shown to have highly reliable properties when used among people with epilepsy ^11^. A higher score (on a 0-100 scale) indicates greater QOL. A score of 50 or less on the Physical Component Summary (PCS-12) indicates the presence of a physical condition, while a score of 42 or less on the Mental Component Summary (MCS-12) scores is suggestive of clinical depression ^9^.

### Statistical analysis

Descriptive statistics were used to summarize long-term outcomes among this under-researched cohort of patients. Univariate inferential statistical analyses were conducted to explore: i) associations between clinical and personal demographics as independent variables against ultimate epilepsy diagnosis as a dependent variable; ii) associations between clinical and personal demographics as independent variables against QOL as a dependent variable. Chi-squared analysis, one-way analysis of variance (ANOVA) and Spearman’s correlation analysis were used to assess the significance of relationships between independent and dependent variables. Data were analyzed using SPSS Statistics® Version 28.

### Bias

There was potential for positive response or social desirability bias in relation to interviewees’ self-reports of their QOL. This implicit bias was addressed by the use of a validated QOL measure and a neutral, non-reactive verbal approach by the researcher who conducted the phone-based survey.

## Results

### Recruitment

**Figure 1.**
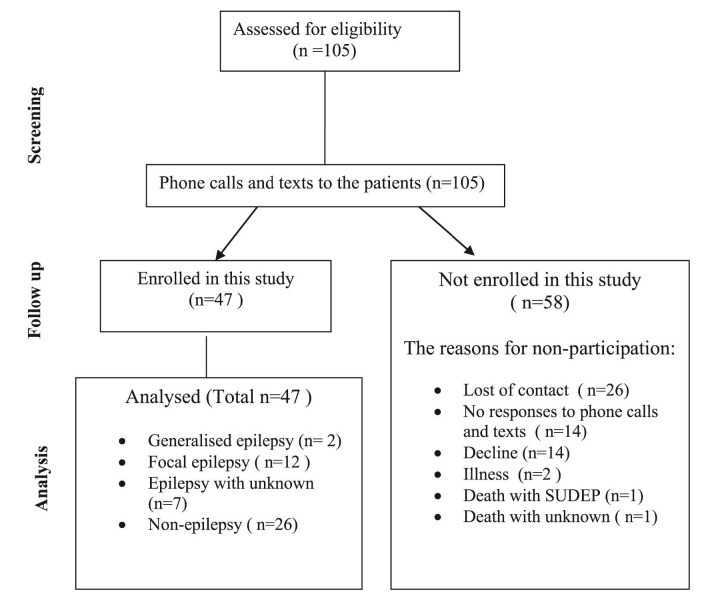
Recruitment diagram. SUDEP, sudden unexpected death in epilepsy.

A total of 47 (n=47) participants were enrolled in the study from June to November 2021 ( Figure 1). This represented a 45% (n=47) response rate from a list of 105 potential participants who participated in the original study over 10 years ago. The clinical features of participants with epilepsy (PWE) (n=21) were generalized epilepsy (n=2), focal epilepsy (n=12), epilepsy with unknown classification (n=7), and 26 patients (55%) who were not diagnosed with epilepsy. Non-response was usually related to the inability to make contact ( *i.e.*, the person had moved house, changed phone number or did not call back). In 13% of cases (14/105), participants declined to participate in the study following initial contact. Overall, 25% (26/105) were lost to contact with the service, and a further 13% (14/105) did not respond to repeated phone contact attempts. Two deaths were reported by family members upon contact. One was a sudden unexpected death in epilepsy (SUDEP), and one was a death of unknown cause.

### Participants' characteristics

The characteristics of the study sample are summarized in Table 1. The majority of the participants were female (n=35, 72%). In total, 21 (45%) patients self-reported being diagnosed with epilepsy. The mean age of participants was 49 years old (range 29–73 years old), and there was no age restriction to participate in this study. Most participants (79%) were exclusively English speakers, and 21% spoke a language other than English at home. The majority were in a marriage-type relationship (66%). A majority held a current driving license (87%). Most took ASM at some point (70%). Among these, 64% had discontinued them at the time of the interview. In terms of QOL, the mean PCS-12 score was 47.4 (SD 12.5), and the mean for the MCS-12 was 49.9 (SD 10.9).

**Table 1.** Participant characteristics.

Characteristics	Results (n=47)
***Personal demographics*:**	
**Age**, years	Mean 49.0; SD 12.9
Sex	Male: Female Ratio: 28:72
**Culturally/linguistically diverse**	Yes: No Ratio: 21:79
I **n a marriage-type relationship**	Yes: No Ratio: 66:34
**In full-time employment**	Yes: No Ratio: 47:53
**Completed higher education (Bachelor's degree or** higher **)**	Yes: No Ratio: 45:55
**Hold a current driver's license**	Yes: No Ratio: 87:13
**Receiving government benefits**	Yes: No Ratio: 43:57
***Clinical demographics*:**	
**Diagnosis**	Epilepsy: Not epilepsy Ratio: 45:55
**Experienced a seizure event from 2007 to 2021**	Yes: No Ratio: 51:49
**Currently taking anti-seizure medication**	Yes: No Ratio: 36:64
**Taken an anti-seizure medication from 2007 to 2021**	Yes: No Ratio: 70:30
***Quality of life:***	
**SF-12 v2 (Physical)**	Mean 47.4 (SD 12.5)
**SF-12 v2 (Mental)**	Mean 49.9 (SD 10.9)

### Epilepsy diagnosis

Overall, 21 participants (45 %; 21/47) retained or ultimately received a diagnosis of epilepsy at follow-up in 2021. Nine participants (19%; 9/47) reported that their diagnosis had been changed from an original epilepsy diagnosis to a non-epilepsy diagnosis. These diagnoses were: single seizure only (n=2), migraine (n=2), neuralgia (n=2), vasovagal syncope (n=1), over-reactive bladder (n=1) and psychogenic non-epileptic seizure (n=1). The remaining 17 participants (36%; 17/47) had unknown diagnoses.

Among the PWE, 21 (n=21; 45%) reported that they were diagnosed with epilepsy (focal, generalized or unknown classification) before they had their AEEG tests between 2007 and 2010. The age of seizure onset at baseline was from 1–58 years old. Four participants reported that their diagnosis was changed to epilepsy during the period between the completion of epilepsy investigations in 2007/10 and follow-up in 2021. A total of 13 of the 21 PWE (62%) reported that they had been taking their ASMs throughout this period, and eight (38%) reported discontinuing their ASMs at the time of follow-up. Three of these eight participants reported stopping taking their ASMs in response to their doctor's decision because of the cessation of seizures. The remaining five reported deciding to self-terminate their ASMs due to perceived ineffectiveness. This group (n=5) indicated that they had suffered from seizure events, and they had decided to focus on lifestyle changes and coping skills to manage their seizures and their health.

Table 2 summarizes significant cross-sectional relationships between participant characteristics and epilepsy diagnosis. PWE had significantly higher education levels (p=.006), were less likely to be in a marriage-type relationship (p=.02), less likely to hold a current driver's license (p=.04), more likely to have experienced a seizure event at some point during the follow-up period (p=.01) and more likely to have taken ASM during the follow-up period (p=.007), than those without an epilepsy diagnosis.

**Table 2.** Significant cross-sectional relationships between participant descriptors and the presence of an epilepsy diagnosis.

Descriptor	Difference located	Test result (P)
**In a marriage-type relationship**	Epilepsy diagnosis: 47.6% = Yes. No epilepsy diagnosis: 80.8% = Yes	x ^2^=5.7 (P=0.02)
**Completed bachelor's degree or higher**	Epilepsy diagnosis: 66.7% = Yes. No epilepsy diagnosis: 26.9% = Yes	x ^2^=7.4 (P=0.006)
**Hold a current driver's license**	Epilepsy diagnosis: 76.2% = Yes. No epilepsy diagnosis: 96.2% = Yes	x ^2^=4.2 (P=0.04)
**Experienced a seizure event from 2007 to** **2021**	Epilepsy diagnosis: 71.4% = Yes. No epilepsy diagnosis: 34.6% = Yes	x ^2^=6.3 (P=0.01)
**Taken anti-seizure medication from 2007 to** **2021**	Epilepsy diagnosis: 100% = Yes. No epilepsy diagnosis: 46.2% = Yes	x ^2^=16.1 (P<0.001)
**Currently taking anti-seizure medication**	Epilepsy diagnosis: 57.1% = Yes. No epilepsy diagnosis: 19.2% = Yes	x ^2^=7.2 (P=0.007)

### Quality of life (QOL)

Applying the cut-off values (a score of <50 PCS-12,< 42 MCS-12) to our dataset suggested that 47% of participants had a physical condition (in QOL terms), and 19% of participants might have had clinical depression at the time of the survey. No significant cross-sectional relationships were located for SF-12 v2 (MCS-12). Table 3 summarizes significant cross-sectional relationships by SF-12 v2 (PCS-12). Older age, not being in full-time employment, not being in receipt of government benefits and experiencing a seizure event at some point during the follow-up period were all associated with lower physical QOL. A cross-sectional assessment of the three outcome variables of interest against each other revealed no significant relationships.

**Table 3.** Significant cross-sectional relationships between participant descriptors and SF-12 v2 (Physical) score.

Descriptor	SF-12 v2 (PCS-12)	Test Result (P)
**Age,** years	Significant negative correlation (Spearman)	Rho= -0.46 (P=0.03)
**In full-time employment**	Yes - Mean score: 52.6 No - Mean score: 42.7	ANOVA: F=8.4 (P=0.006)
**Receiving government benefits**	Yes - Mean score: 50.6 No - Mean score: 42.9	ANOVA: F=4.7 (P=0.04)
**Experienced a seizure event** between **2010 & 2021**	Yes - Mean score: 43.8 No - Mean score: 51.0	ANOVA: F=4.2 (P=0.047)

## Discussion

A key finding from this 12-year follow-up study relates to epilepsy diagnosis at follow-up. Prolonged EEG studies aim to capture any epileptiform features to provide electrophysiological support for a clinical diagnosis of epilepsy. Nearly half of the participants in this study who did not have interictal epileptiform discharges (IEDs) on their original EEG studies remained diagnosed with epilepsy and took ASMs at follow-up. The limitations of scalp EEG recording, the effectiveness of ASMs and the cyclic nature of seizure patterns are possible reasons that this group did not have paroxysmal events or epileptiform discharges at the time of EEG recording.

Scalp EEG recording has limitations in detecting all IEDs. Scalp EEG electrodes can sample only one-third of the cortex and cannot detect any discharges within the sulci, basal regions, interhemispheric regions, and deep cortex ^14^. The mechanisms underlying IED generation are still unknown, although it is generally accepted that IEDs reflect cortical hyperexcitability ^15^. The presence of IEDs supports the establishment of a clinical diagnosis of epilepsy, forms part of the characterization of epilepsy syndromes and aids in localizing the epileptogenic region in patients with focal epilepsy. With regards to the effectiveness of the ASM as a driver of this result, many ASMs, particularly lamotrigine, valproate (VPA) and levetiracetam, reduce or suppress the occurrence of IEDs ^14^. Overall, 10 PWE had taken either lamotrigine or levetiracetam during their original ambulatory EEG test. This could be expected to reduce the frequency of both paroxysmal clinical events as well as suppress IEDs. Patients with longer-duration cyclic patterns of seizure occurrence may have undergone their AEEG testing during a period of relative quiescence, thus reducing the sensitivity of the test in recording paroxysmal events. There is increasing interest in ultra-long-term EEG recording, which could be expected to improve diagnostic sensitivity in patients with long cyclic seizure frequency variations ^16, 17^. All PWE (n=21) had tried taking ASM at some point during the follow-up period, and 13/21 (57%) were taking ASMs at the follow-up time. Six were still taking a single ASM as monotherapy with either carbamazepine or levetiracetam. Seven PWE took two or more ASMs. The ASMs were carbamazepine (n=4), lamotrigine (n=6), levetiracetam (n=6), VPA (n= 1), lacosamide (n=1), perampanel (n=1), clonazepam (n=1), diazepam (n=1) and clobazam (n=1). Some of the participants without epilepsy also took ASMs for their medical conditions, such as trigeminal neuralgia, migraines and panic attack. These were topiramate (n=2), carbamazepine (n=1) and diazepam (n=1). The ASM, VPA (n=7) was reported as having ceased due to no recurrent seizures/events (n=6) and medication ineffectiveness (n=1).

The finding with regards to VPA prescription among people without an epilepsy diagnosis suggested a medical professional's preference to prescribe VPA to treat the patient with unknown/unclassified seizures. VPA has been effective in focal and generalized seizures and used as monotherapy and adjunctive therapy for focal impaired awareness seizure for many years ^18^. In the 1970s, VPA was recommended as the first line of therapy because it abolished photosensitivity and epileptiform interictal discharges on EEG and atypical absence seizures ^19^. VPA was recommended as the first choice of ASM in treating seizures from 1989–2009 ^19^. The SANAD study demonstrated the effectiveness of VPA over lamotrigine and topiramate in patients with generalized or unclassified seizures ^20^. Against such findings, VPA incurs a high risk of teratogenicity, causing physical malformation and has been linked with neurodevelopmental delay ^21– 23^. In our study, a small number of participants were treated with their events or seizures with VPA. Among these, five were women of childbearing age. Medical prescribers might not be aware of teratogenicity risks and might prefer to prescribe VPA to treat them as unknown/unclassified seizures. One of the first studies focused on the relationship between VPA and teratogenicity, and VPA prescription trends were assessed during 2008–2013 ^24^. The rate of VPA prescription to women aged 15–44 years declined slightly from 0.35% in 2008 to 0.31% in 2013. This declining trend was significant for women with epilepsy. The data from the Australian Register of ASMs in Pregnancy for the period 1999–2007 showed that VPA prescription to pregnant women decreased over time ^25^. VPA is still used for childbearing-aged women with epilepsy if no effective ASM is available ^26^.

The study by Faulkner *et al.*, ^7^ did not measure the QOL of the participants. The QOL-specific results in this study present a mixed picture. The high proportion of participants who scored below the cut-off for the presence of a physical condition suggests that PWE in the sample regularly experienced physical discomfort. Older age, not being in full-time employment, not being in receipt of government benefits and experiencing a seizure event at some point during the follow-up period were all associated with lower physical QOL in our study. Older age, lower educational level, comorbid anxiety and depression and non-adherence to drugs have been associated with poor quality of life among PWE in prior studies ^27^. Our study found no significant cross-sectional relationships between participant characteristics and mental QOL scoring. This may be related to the small sample size obtained in the study.

In addition to QOL, one of two death-related exclusions from this long-term follow-up study was a SUDEP. Better recognition of the relationships between seizure activity, interictal changes, and other physiological cycles, such as cardio-pulmonary cycles, may, in the future, help clarify the reasons for SUDEP. The incidence of SUDEP increases to about 1–6 per 1,000 per year in the chronic epilepsy population ^26^. This participant did not have an epilepsy diagnosis during the AEEG test. However, the participant was diagnosed with epilepsy by a professional healthcare provider and suffered from seizures throughout their life.

Our study results indicated that PWE were less likely to have spousal partnerships, which could be due to the stigma and social exclusion that people with epilepsy often face in different cultures. Social support from marriage has a positive impact on health ^28^. People with epilepsy who reported a lack of affectionate support have been found to be likelier to report poor QOL ^29^. Partnered persons report better psychological and physical health compared to those who are not partnered ^30^. Previous epidemiological surveys have consistently found that people with epilepsy are more likely to report never being partnered than those without epilepsy ^28^. Moreover, unpartnered people with epilepsy have a poorer QOL than partnered people with epilepsy, as shown in studies comparing PWE with individuals with other chronic diseases or the general population ^31^. This has also been shown in studies comparing PWE with individuals with other chronic diseases or the general population ^31– 33^.

## Conclusions

It is crucial to take an accurate history and perform a neurological examination when diagnosing epilepsy, including assessing the risk of SUDEP. EEG testing is a supplementary tool to support clinical assessment. Emphasizing the importance of a thorough clinical examination, including a good eyewitness account and EEG testing, can help prevent misdiagnosis. Prolonged AEEG is a vital tool to support the diagnostic evaluation of individuals suspected to have epilepsy. Our long-term follow-up of patients who underwent prolonged AEEG tests found that nearly half ultimately received a diagnosis of epilepsy despite negative initial test results. This suggests that clinical assessment is just as important as electrophysiologic assessment in establishing a diagnosis of epilepsy.

### Limitations

The long length of follow-up time was both an advantage to the study (in that it reflects a long and meaningful follow-up period) and a disadvantage in that loss to follow-up was inevitable and may have introduced selection bias to the study. The diagnosis of epilepsy at follow-up was based on the participant's self-report and not verified clinically. Finally, the sample size was modest.

## Data Availability

The datasets utilized in this research are not accessible to the public due to ethical limitations imposed by the local health district. Nevertheless, interested individuals may request access to the data from the corresponding author, Sumika Ouchida, who will provide assistance with the results of the study.
