# Supplementary material for: The long-term outcomes of patients with negative prolonged ambulatory electroencephalography tests: A cross-sectional follow-up study
Source: Health Open Res. 2023 Aug 10;5:26. [Version 1] doi: 10.12688/healthopenres.13351.1 (PMC11065127; doi:10.12688/healthopenres.13351.1)
Supplement: Peer review history

# Reviewer report: Reviewer response for version 1

**DOI:** 10.21956/healthopenres.14435.r27532

**Published:** 2023-11-23

**Authors:** Enrica Bonanni

**Affiliations:** 1 University of Pisa, Pisa, Italy

The authors performed a very interesting and well written study, rightly identifying the main strenghts and limitations of their work. As the authors remark, recording (or failing to record) an IED on scalp EEG cannot be the sole means by which a diagnosis of epilepsy is made nor ruled out, and clinical judgement should help lead to a correct diagnosis. Indeed, it would be interesting, either in this study or in a follow-up study, to understand which factors allowed a diagnosis of epilepsy to finally be made in those who reported having received it.

Is the work clearly and accurately presented and does it cite the current literature?

Yes

If applicable, is the statistical analysis and its interpretation appropriate?

Yes

Are all the source data underlying the results available to ensure full reproducibility?

No

Is the study design appropriate and is the work technically sound?

Yes

Are the conclusions drawn adequately supported by the results?

Yes

Are sufficient details of methods and analysis provided to allow replication by others?

Yes

Reviewer Expertise:

Clinical neurology, electroencephalography, epilepsy, sleep medicine

I confirm that I have read this submission and believe that I have an appropriate level of expertise to confirm that it is of an acceptable scientific standard.

# Response

**Published:** 2023-12-01

**Authors:** Sumika Ouchida

**Affiliations:** Neurology, Royal Prince Alfred Hospital, Camperdown, New South Wales, Australia

Thank you for sharing your feedback with us. In our study, we asked patients a simple question: "Have you been diagnosed with epilepsy and if yes, what types of epilepsy were you diagnosed with?" The aim of the study was to describe the outcomes of patients who had negative EEG tests over ten years ago. It is important to note that we did not clinically diagnose the patients with epilepsy or identify epilepsy syndromes. However, some patients with focal epilepsy reported experiencing a "Deja Vu" feeling before the onset of their seizures. In future studies, we plan to collect data on seizure characteristics to provide a more comprehensive analysis of patients' conditions.

# Reviewer report: Reviewer response for version 1

**DOI:** 10.21956/healthopenres.14435.r27528

**Published:** 2023-11-23

**Authors:** Radhakrishnan Delhibabu

**Affiliations:** 1 VIT Vellore, Tamil Nadu, India

I found the paper to be quite interesting. Your research highlights the challenges that AEEG still faces in replacing or significantly complementing regular EEG. The findings you presented shed light on the long road ahead for AEEG in terms of its clinical application and reliability. I appreciate the thoroughness of your study and the insights it provides to the scientific community. Finally, It has not reached the levels of Holter.

Is the work clearly and accurately presented and does it cite the current literature?

Yes

If applicable, is the statistical analysis and its interpretation appropriate?

Yes

Are all the source data underlying the results available to ensure full reproducibility?

Yes

Is the study design appropriate and is the work technically sound?

Yes

Are the conclusions drawn adequately supported by the results?

Yes

Are sufficient details of methods and analysis provided to allow replication by others?

Yes

Reviewer Expertise:

Noise removal methods on ambulatory EEG, Brain-Computer Interface, Cognitive Modeling, AI&ML

I confirm that I have read this submission and believe that I have an appropriate level of expertise to confirm that it is of an acceptable scientific standard.

# Response

**Published:** 2023-12-01

**Authors:** Sumika Ouchida

**Affiliations:** Neurology, Royal Prince Alfred Hospital, Camperdown, New South Wales, Australia

Thank you for your feedback. Recording paroxysmal events without antiseizure medication during ambulatory EEG tests can be difficult. Although inpatient video EEG monitoring is still the preferred option for patients who require epilepsy surgery, ambulatory EEG tests are an excellent choice for patients seeking a clinical diagnosis. Inpatient video EEG monitoring is a very expensive and time-consuming procedure, whereas the ambulatory EEG test is more cost-effective and patient-centred. Our healthcare system needs to shift from hospital-oriented care to community-based care. In the near future, we anticipate the development of an implantable recording device similar to a cardiac implantable loop recorder. This device will provide more information and increase the number of clinical diagnoses for patients.
